# Genetic variation in humoral response to an *Escherichia coli* O157:H7 vaccine in beef cattle

**DOI:** 10.1371/journal.pone.0197347

**Published:** 2018-05-14

**Authors:** Kara B. Marley, Larry A. Kuehn, John W. Keele, Benjamin W. Wileman, Michael G. Gonda

**Affiliations:** 1 Department of Animal Science, South Dakota State University, Brookings, South Dakota, United States of America; 2 Meat Animal Research Center, United States Department of Agriculture, Clay Center, Nebraska, United States of America; 3 Department of Animal Sciences and Industry, Kansas State University, Manhattan, Kansas, United States of America; Universitat de Lleida, SPAIN

## Abstract

Individuals often respond differently to the same vaccine; some of this variation may be caused by genetic differences among animals. Our objective was to estimate heritability and identify genomic regions associated with humoral response to an *Escherichia coli* O157:H7 vaccine in beef cattle. Crossbred beef cattle (n = 651) were vaccinated with a commercially available *E*. *coli* O157:H7 vaccine. Serum was collected at time of initial vaccination (d 0), booster (d 21), and d 56 after initial vaccination. Total antibodies specific to siderophore receptor and porin proteins in the vaccine were quantified by enzyme-linked immunosorbent assay. Genomic DNA was isolated from whole blood and genotyped with the bovine GeneSeek Genomic Profiler-High Density 78K or 26K Single Nucleotide Polymorphism BeadChip and imputed to 777,000 SNP genotypes. Heritability was estimated by restricted maximum likelihood (REML) using both 1) pedigree and 2) genomic relationships among individuals. Fixed effects were contemporary group, calf age, sex, principal components from SNP genotype data, and pedigree-derived heterozygosity effects. Additive and dominance effects of SNPs were estimated individually while accounting for contemporary group, sex, and the top 20 principal components calculated from the genomic relationship matrix. Heritability of initial response to vaccination (d 21 –d 0) was 0.10 ± 0.175 using pedigree relationships and 0.14 ± 0.149 using genomic relationships, but neither estimate was statistically different from zero. Heritability of booster (d 56 –d 21) and overall (d 56 –d 0) responses were low and not statistically significant from zero. There were no clusters of linked SNP associated with vaccine response, but eight regionally isolated SNPs were significantly associated with initial or overall response to vaccination. Regional genetic variation for initial response to an *E*. *coli* O157:H7 vaccine was observed, although overall heritability of this response was not statistically significant from zero.

## Introduction

Responses to vaccination vary among individuals and genetic differences clearly contribute to variation in vaccine response in human populations [1, 2]. However, comparatively few studies have investigated genetic variation in livestock to vaccine response. In mammalian livestock species, most of these studies have focused on cattle, specifically foot-and-mouth disease virus (FMDV) and Bovine Respiratory Disease (BRD) vaccines. Humoral response to a Bovine Respiratory Syncytial Virus (BRSV) vaccine was reported to be moderately heritable (h^2^) [3], while changes in blood leukocyte subpopulations after vaccination for BRD pathogens were also reported to be heritable and associated with binary health scores (0 = healthy; 1 = ill) [4]. Quantitative trait loci (QTL) have been discovered for response to both a FMDV peptide vaccine [5] and BRSV vaccine [6, 7]. Furthermore, the Major Histocompatibility Complex alleles have been associated with response to a FMDV peptide virus vaccine [8, 9].

Only one of these studies estimated heritability of humoral vaccine response in cattle [3]. A need exists to determine if heritability estimates for humoral vaccine response are consistent regardless of vaccine used or if vaccine type affects these estimates. To address this need, heritability of humoral vaccine response over a wider range of vaccines needs to be determined. Further, previous studies have mapped QTL using F_2_ and backcross populations, which have too much linkage disequilibrium (LD) to support fine mapping causative polymorphisms underlying the QTL. Genome-wide association studies, made possible by the development of single nucleotide polymorphism (SNP) genotyping platforms, can more narrowly fine map locations of causative polymorphisms underlying QTL as long as sample size is adequate to offset the increased statistical threshold required because of multiple testing of thousands of SNP.

*Escherichia coli* O157:H7 is a bacteria that does not cause clinical morbidity or mortality in cattle but is a source of foodborne illness to humans. Humans become infected with *E*. *coli* O157:H7 through ingestion of contaminated beef products; cattle shedding this bacterium in their feces are the main source of contamination. A vaccine is commercially available that can decrease fecal shedding of *E*. *coli* O157:H7 [10]. Our objectives were to determine whether humoral response to this *E*. *coli* O157:H7 vaccine is heritable and to identify genomic regions associated with this humoral response.

## Materials and methods

### Experimental design

Our *a priori* hypotheses were that humoral response to an *E*. *coli* O157:H7 vaccine would be heritable and that genomic regions associated with humoral response to this vaccine could be identified. This vaccine contains purified siderophore receptor and porin (SRP) proteins derived from bacterial extracts of *E*. *coli* O157:H7. These receptors are necessary for uptake of iron by *E*. *coli* O157:H7 cells. This vaccine is designed to generate antibodies specific to these SRP proteins, which then bind to SRPs on *E*. *coli* O157:H7. The antibodies block entry of iron into *E*. *coli* O157:H7, causing cell death. A cohort study was designed where 651 calves were administered this *E*. *coli* O157:H7 vaccine and humoral response to vaccination was measured. The vaccine was administered twice: at initiation of the trial (d 0) and 21 d after initial vaccine administration (booster). Total serum antibodies specific to SRP proteins were quantified at three time points: initial vaccination (d 0), 21 d post-vaccination (booster administration), and 56 d post-vaccination. Humoral response to vaccination was then assessed by subtracting d 0 antibodies from antibodies present at d 21 (“initial response”) and d 56 (“overall response”). Humoral response to booster vaccination was also determined by subtracting d 21 antibodies from d 56 antibodies (“booster response”). A blood sample was collected from calves for DNA isolation and DNA was genotyped with the GeneSeek Genomic Profiler-High Density (GGP-HD) or low density (GGP-LD) SNP BeadChip. The SNP genotypes were used to estimate genomic relationships among calves and test for associations between genotypes and vaccine response.

### Animals

Research with animals involved in this study was approved by the USDA-ARS-Meat Animal Research Center Institutional Animal Care and Use Committee. Calves (*n* = 672) from the USDA-Meat Animal Research Center Germplasm Evaluation (GPE) Program [11] were sampled. The goal of the GPE Program is to maintain a herd of cattle representative of germplasm in the US beef industry for the purpose of estimating breed and heterosis effects. This crossbred herd includes germplasm from 16 of the most common beef cattle breeds and composites in the USA: Angus, Beefmaster, Brahman, Brangus, Braunvieh, Charolais, Chiangus, Gelbvieh, Hereford, Limousin, Maine Anjou, Red Angus, Salers, Santa Gertrudis, Shorthorn, and Simmental. The calves were produced from purebred, F1, and F1 x F1 dams (not F2 as the parents of these dams did not have to be the same breed) crossed to F1 and purebred sires. Initial crosses (F1) of the dams were based on AI matings of industry relevant sires (from the 16 breeds mentioned) from each breed to Angus, Hereford, Charolais, Simmental, or composite cows. The bulls were also derived using AI matings from industry relevant sires. Thus, this group of crossbred animals was highly admixed but would still be expected to have large chromosomal segments representing their purebred origins that were 3 or fewer generations removed.

Statistical power and precision for estimating heritability are primarily driven by variance in relationship between animals [12]. Our population structure is expected to be 3 to 4 times more powerful than a comparably sized population of 93 unrelated half-sib families with an equal number of 7 progeny per family. Number of progeny per sire varied between 1 and 37 with an average of 5.03 progeny per sire. Two hundred eighty-eight calves were in 16 half-sibships comprised of 10–37 progeny per sire; 154 were in 39 half-sibships comprised of 2–9 progeny per sire; and 41 calves were in single sire families. The variance in relationship coefficient between calves was 0.00175 for pedigree-based relationships and 0.00206 for genomic relationships indicating that the genomic relationship had slightly more power. For comparison, 651 calves in 93 half-sibships with 7 progeny for each sire would have a variance in relationship of 0.00057. The variance in relationship was computed as the variance among the 211,575 lower triangular off-diagonal elements. However, matrix methods are not necessary because a half-sib family structure is simple. The off-diagonals of the relationship matrix are comprised of only 2 values .25 for half-sib pairs and 0 for unrelated animals. There would be 21 = (7 * 6 / 2) pairs of half sibs in each family and there are 93 families; hence, 1,953 half-sib pairs total each with a relationship of 0.25. The remaining pairs (n = 209,622 = 651 * 650 / 2–1,953) are unrelated so their relationship is 0. The mean relationship in this half-sib pedigree would be 0.0023 = (0.25 * 1,953 + 0 * 209,622) / (1,953 + 209,622). Consequently, the variance would be 0.00057 = [(.25–.0023)^2^ * 1,953 + (0–0.0023)^2^ * 209,622] / (1,953 + 209,622). Calves (both sexes) were born between March and June 2012 and averaged 127 ± 17 d of age at time of initial vaccination. Dams of the calves averaged 6.8 ± 3.23 years of age. Dams and calves were not administered an *E*. *coli* O157:H7 vaccine prior to this study. Calves and their dams were raised at four locations separated by at least 2 miles on 50 square miles of federal property at USDA-MARC located ~4 miles west of Clay Center, NE.

### Vaccination and sampling

Calves were vaccinated subcutaneously with a commercially available *E*. *coli* O157:H7 vaccine (Zoetis Animal Health) following the manufacturer’s label instructions. A booster shot was administered 21 d following initial vaccination. Approximately 10 mL blood was collected from the jugular vein into serum vacutainer tubes at three time points: 1) time of initial vaccination (d 0), 2) time of booster vaccination (d 21), and 3) 56 d after initial vaccination. Blood was centrifuged and sera collected and frozen at -80 °C prior to antibody quantification. A fourth blood sample (10 mL) was also collected into syringes with 40% Ethylenediaminetetraacetic Acid (EDTA) for DNA isolation.

### Antibody measurements

Antibodies in sera samples were measured by indirect enzyme-linked immunosorbent assay (ELISA) in parallel with positive and negative controls as previously described [13]. Individual serum samples were measured in duplicate and averaged. The positive control was serum collected from a calf hyper-immunized with *E*. *coli* O157 SRP antigen. Sample-to-positive (*S/P*) ratios for each individual were calculated from optical densities as follows:

$$S/P\;=\;\frac{{OD}_{S}-{OD}_{N}}{{OD}_{P}-{OD}_{N}}$$

where *OD*_*S*_ = optical density of individual sera samples, *OD*_*N*_ = optical density of negative control, and *OD*_*P*_ = optical density of positive control. The S/P ratios were used as our measure of antibodies at each time point for genetic analyses.

### SNP genotyping

Blood samples were used to extract DNA using the BioSprint 96 DNA Blood Kit or QIAamp DNA Mini Kit (Qiagen; Valencia, CA). The DNA samples were genotyped with the bovine GeneSeek GGP-HD or LD SNP BeadChip by Neogen Corporation (Lincoln, NE). This BeadChip is based on Illumina Infinium technology, which simultaneously genotypes approximately 78,000 or 26,000 SNPs, respectively, in a DNA sample by primer extension in a single reaction.

### Heritability estimation

Heritability was estimated for six traits: “initial response”, “booster response”, and “overall response” as defined in the experimental design section and antibodies present at 0 (time of initial vaccination), 21 (time of booster vaccination), and 56 days post-initial vaccination. Heritability was estimated by 2 different methods using pedigree relationships among animals by Multiple Trait Derivative Free Restricted Maximum Likelihood (MTDF-REML) with SAS software (SAS Institute, Cary, NC) and using genomic relationships with optim() of R with method = “L-BFGS-B”. These models included fixed effects of birth location (contemporary group) and gender and covariates of calf age and pedigree derived expected heterozygosity as well as the top 20 principal components from the SNP genotype data. The principal components analysis was completed to account for possible unknown genetic structure within our sample. The models were designed as follows:

Y=Xb+Za+e

where *Y* was vector of phenotypes, *b* was vector of fixed effects, *a* was vector of random additive animal effects, *e* was vector of residuals, and *X* and *Z* were incidence matrices relating vectors *a* and *b* to phenotypes. Variance of random additive animal effects was estimated by $A\sigma_{a}^{2}$ where *A* was either the pedigree or genomic relationship matrix and $\sigma_{a}^{2}$ was additive genetic variance. Variance of residuals was estimated by $I\sigma_{e}^{2}$ where *I* was the identity matrix and $\sigma_{e}^{2}$ was residual variance. Variance components for each of the six traits were estimated separately.

### Genome-wide association analysis (GWAA)

A total of 21 calves were removed from the study because genotype quality was insufficient (< 85% call rate). Cattle were imputed to 777K SNP density from Bovine GGP 77K for 502 animals and GGP 20K for 149 animals. In addition to the medium and low SNP data (GGP 78K and GGP20K genotypes), imputing was based on high density data (Bovine HD or GGPF250 genotypes) on parents, grandparents, and a few siblings of the animals phenotyped in our study. Including SNPs from high density panels on close relatives as we have in this study has led to accurate (95%) imputing in progeny with low density genotypes in previous studies [14]. We used FindHap [14, 15] to impute taking into account pedigree and high density genotypes on relatives.

A GWAA was completed on six traits: the “initial response”, “booster response”, and “overall response” defined previously and *E*. *coli* O157 antibodies present at 0 (time of initial vaccination), 21 (time of booster vaccination), and 56 days post-vaccination. The model used for GWAA was the same as for estimating heritability with the exception that genotypes for individual SNP coded as 0, 1 or 2 copies of the B allele were augmented to the X matrix one SNP at a time. For this analysis, a common heritability equal to heritability for each trait for all SNPs was used to reduce computational load. The genome-wide significance and suggestive levels were achieved when the nominal *P* = 5 x 10^−8^ and 1 x 10^−5^, respectively. Manhattan plots were created with the R package “qqman” [16] by plotting the -log_10_(P-value) by the chromosome and position of each SNP. Results from this study have been uploaded to the Animal QTL database (https://www.animalgenome.org/cgi-bin/QTLdb/index). To refine effects for the statistically most significant 100 SNPs for each trait, a simultaneous analysis was completed that allowed SNPs to change heritability and environmental variance.

## Results

The vaccine was effective at eliciting a humoral response specific for *E*. *coli* O157:H7 SRP proteins (Table 1). Most calves (99%) developed a humoral response to the initial administration of vaccine by d 21, although responses (S/P ratios d 21 –d 0) ranged from 0 to 0.98. After booster vaccination, *E*. *coli* O157-specific antibodies were present in sera of all calves by d 56 and humoral response was less variable.

**Table 1:** Humoral response to vaccination with *E*. *coli* O157:H7 Siderophore and Porin Receptor (SRP) proteins as measured by an ELISA specific for *E*. *coli* O157:H7 SRPs (*n* = 651).

Humoral Response Phenotypes	Mean S/P Ratios ± SD
Antibodies at d 0	0.085 ± 0.124
Antibodies at d 21	0.647 ± 0.169
Antibodies at d 56	0.853 ± 0.103
Initial Response (d 21 –d 0)	0.562 ± 0.206
Booster Response (d 56 –d 21)	0.206 ± 0.161
Overall Response (d 56 –d 0)	0.768 ± 0.161

Different heritability estimates were obtained depending on the trait analyzed. Using MTDF-REML and a pedigree relationship matrix with breed effects, none of the traits exhibited a heritability estimate different from zero (Table 2). Amount of *E*. *coli* O157 antibodies at day 21 (h^2^ = 0.14 ± 0.179) and the initial response (d 21 –d 0; h^2^ = 0.10 ± 0.175) traits had the highest heritability estimates. Our choice of relationship matrix (pedigree or genomic) had little effect on variance component estimation; variance component and heritability estimates were similar regardless of whether a pedigree or genomic relationship matrix was used, with the exception of antibodies present at day 21 (h^2^ = 0.23 ± 0.152 with genomic matrix; Table 2). Birth location (contemporary group) and sex were significantly associated with most traits (Table 3). Sex was associated with all traits except d 21 antibody concentration and steers had higher antibody levels and elicited a stronger humoral immune response than heifers. Age at vaccination was significantly associated with initial and overall response to vaccination, as well as antibodies present at day 0. Older animals elicited a stronger humoral initial and overall response and had fewer antibodies present at day 0. Principal components and pedigree-based expected heterozygosity were not significantly associated with any traits.

**Table 2:** Heritability estimates for humoral response to *E*. *coli* O157:H7 Siderophore and Receptor Porin (SRP) protein vaccination in calves (*n* = 651).

Trait	Method of Calculating Relationships ^a^	Heritability	Standard Error	*P*-value
Antibodies at day 0	Pedigree	0.0011	0.1594	0.994
	Genomic	0	0.1416	1.000
Antibodies at day 21	Pedigree	0.1402	0.1791	0.434
	Genomic	0.2270	0.1516	0.134
Antibodies at day 56	Pedigree	0	0.1592	1.000
	Genomic	0.0085	0.1422	0.953
Initial Response ^b^	Pedigree	0.1038	0.1746	0.552
	Genomic	0.1387	0.1494	0.353
Booster Response ^b^	Pedigree	0.0614	0.1687	0.716
	Genomic	0	0.1416	1.000
Overall Response ^b^	Pedigree	0	0.1592	1.000
	Genomic	0	0.1416	1.000

^a^ Data used to estimate covariance coefficients among individuals: pedigree or genomic

^b^ Initial Response = Antibodies present at d 21 minus d 0 (time of vaccination); Booster Response = Antibodies present at d 56 minus d 21 (time of booster vaccination); Overall Response = Antibodies present at d 56 minus d 0

**Table 3:** Fixed effects and covariates (± SE) for humoral response to *E*. *coli* O157:H7 Siderophore and Receptor Porin (SRP) protein vaccination in calves (*n* = 651).

Fixed Effect or Covariate	Relationship Matrix ^a^	Initial Response ^b^			Booster Response ^b^			Overall Response ^b^		
		Effect	SE	*P*	Effect	SE	*P*	Effect	SE	*P*
Sex	Pedigree	0.0186 ^d^	0.0170	0.275	0.0403 ^d^	0.0145	0.006	0.0594 ^d^	0.0139	< .001
	Genomic	0.0188 ^d^	0.0170	0.271	0.0401 ^d^	0.0145	0.006	0.0594 ^d^	0.0139	< .001
Age ^c^	Pedigree	0.0014	0.0005	0.006	-0.0012	0.0004	0.009	0.0003	0.0004	0.474
	Genomic	0.0014	0.0005	0.007	-0.0012	0.0004	0.007	0.0003	0.0004	0.474

^a^ Data used to estimate covariance coefficients among individuals: pedigree or genomic

^b^ Initial Response = Antibodies present at d 21 minus d 0 (time of vaccination); Booster Response = Antibodies present at d 56 minus d 21 (time of booster vaccination); Overall Response = Antibodies present at d 56 minus d 0

^c^ Age at time of vaccination (months)

^d^ Sex effects are defined as “steers—heifers”; hence, when steers have higher humoral vaccine responses than heifers, effect sizes will be positive

**Fig 1:**
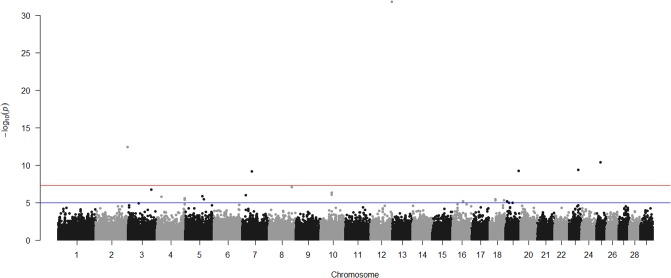
Manhattan plot of genome-wide single nucleotide polymorphisms (SNPs) associated with initial response to vaccination with *E*. *coli* O157:H7 Siderophore and Receptor Porin (SRP) proteins in calves (*n* = 651). Red line = Genome-Wide Significance Level (*P* < 5 x 10^−8^); Blue line = Suggestive Significance Level (*P* < 1 x 10^−5^). A genomic relationship matrix was used for this Manhattan Plot, but similar results were obtained using a pedigree relationship matrix (results not shown).

**Fig 2:**
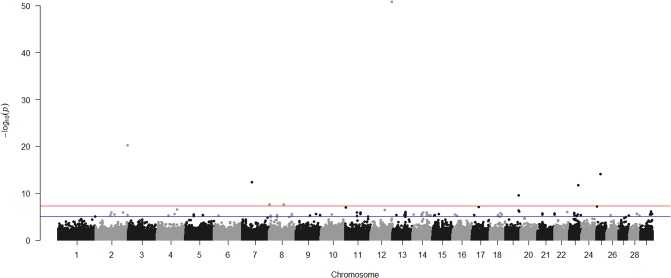
Manhattan plot of genome-wide single nucleotide polymorphisms (SNPs) associated with overall response to vaccination with *E*. *coli* O157:H7 Siderophore and Receptor Porin (SRP) proteins in calves (*n* = 651). Red line = Genome-Wide Significance Level (*P* < 5 x 10^−8^); Blue line = Suggestive Significance Level (*P* < 1 x 10^−5^). A genomic relationship matrix was used for this Manhattan Plot, but similar results were obtained using a pedigree relationship matrix (results not shown).

Our GWAA identified six SNPs significantly associated at a genome-wide level (*P* < 5 x 10^−8^) with initial response to vaccination (Table 4; Fig 1). These same SNP, plus two additional SNP on BTA 8, were significantly associated with overall response to vaccination at a genome-wide level (Table 5; Fig 2). A total of 22 SNPs were associated at a genome-wide level with antibodies present at day 0 (S1 Table). No SNPs were associated at a genome-wide level with the booster response or antibodies present at day 21 or day 56 following initial vaccination (S1 Table; S1–S4 Figs). Using a pedigree- or genomics-based relationship matrix did not change the SNPs associated with traits at a genome-wide significance level and changes in effect sizes of these SNPs was small (data not shown). The simultaneous analysis, which allowed SNP effects to change heritability and environmental variance estimates, also had little impact on effect sizes of the top 100 SNPs associated with each trait (S1 Table).

**Table 4:** Single nucleotide polymorphisms (SNPs) significantly associated with initial response^a^ to *E*. *coli* O157:H7 Siderophore and Porin Receptor (SRP) protein vaccination (*n* = 651 calves; Genome-Wide *P* < 1 x 10^−8^).

SNP	Chromosome location	Additive Effect ^b^	Dominance Effect ^b^	Gene ^c^
rs135467227	12; 88309027	-0.101 ± 0.008	-0.058 ± 0.029	*Myo16*
rs133926855	2; 134692959	0.071 ± 0.010	0.023 ± 0.010	-
rs136430510	25; 185544426	-0.054 ± 0.013	0.034 ± 0.016	*Acsm4*
rs110418859	23; 39405598	-0.075 ± 0.014	0.079 ± 0.013	*Loc534832*
rs137512794	19; 56728310	-0.037 ± 0.017	-0.133 ± 0.021	-
rs42679472	7; 39707997	0.006 ± 0.010	-0.093 ± 0.015	*Hk3*

^a^ Initial Response = *E*. *coli* O157:H7 SRP antibodies present at d 21 minus d 0 (time of initial vaccination).

^b^ Reported in Sample-to-Positive (S/P) ratio units.

^c^ If SNP is located within a known or predicted gene, the gene is reported.

**Table 5:** Single nucleotide polymorphisms (SNPs) significantly associated with overall response^a^ to *E*. *coli* O157:H7 Siderophore and Porin Receptor (SRP) protein vaccination (*n* = 651 calves; Genome-Wide *P* < 1 x 10^−8^).

SNP	Chromosome location	Additive Effect ^b^	Dominance Effect ^b^	Gene ^c^
rs135467227	12; 88309027	-0.102 ± 0.006	-0.044 ± 0.022	*Myo16*
rs133926855	2; 134692959	0.074 ± 0.008	0.021 ± 0.008	-
rs136430510	25; 185544426	-0.054 ± 0.010	0.029 ± 0.009	*Acsm4*
rs42679472	7; 39707997	-0.013 ± 0.008	-0.091 ± 0.012	*Hk3*
rs110418859	23; 39405598	-0.05 ± 0.011	0.079 ± 0.011	*Loc534832*
rs137512794	19; 56728310	-0.034 ± 0.009	-0.108 ± 0.017	-
rs43558286	8; 61752353	-0.008 ± 0.030	-0.121 ± 0.027	*Zcchc7*
rs136519132	8; 2571975	-0.178 ± 0.036	0.188 ± 0.032	-

^a^ Overall Response = *E*. *coli* O157:H7 SRP antibodies present at d 56 minus d 0 (time of initial vaccination).

^b^ Reported in Sample-to-Positive (S/P) ratio units.

^c^ If SNP is located within a known or predicted gene, the gene is reported.

## Discussion

Our findings are somewhat limited by lack of power and limited sample size for complex traits like humoral immune response to vaccination. This lack of power is an ongoing problem because generally individual labs have a difficult time mustering adequate sample size to compensate for the increased statistical stringency required to overcome multiple testing bias. Indeed, in the case of this experiment 651 animals was not sufficient to conclude that our heritability estimates (max 0.23) were different from zero. However, this lack of power and small sample size can later be rectified by combining these data with future experiments in a meta-analysis [17].

Power of our experiment was very low for realistic regional heritability values ranging from 1 to 10%. Power increases with increasing heritability and increasing SNP window size. Paradoxically, as window size shrinks linkage disequilibrium (LD) increases and eigenvalue variation increases of which both increase power [18]. Unfortunately, as window size shrinks the number of windows to be tested increases leading to a reduction in power resulting from correcting for multiple testing.

It is not clear whether our crossbred population improves or decreases statistical power for estimating heritability and SNP effects. Literature indicates that the effect of crossbreeding might be mixed and that adjusting for breed structure using PCA can actually decrease within breed genomic prediction accuracy [19, 20].

Among our measures of vaccine response, only the initial response (d 21 –d 0) and antibodies present at day 21 exhibited evidence of regional genetic variation. However, overall heritability of these measures of vaccine response were not statistically different from zero. Counter to intuition, whole genome heritability does not have to be the sum of regional heritability or variance from individual SNP effects. This is because the regional genomic relationship matrix isn’t necessarily correlated with the overall genomic relationship matrix or the pedigree relationship matrix. We documented that the regional genomic relationship matrices were different from the overall genomic relationship matrix. Two possibilities exist for why our heritability estimates were not statistically significant: 1) our sample size was not sufficiently powered to detect low heritability estimates or 2) humoral response to this vaccine was not heritable. The time points when humoral response was measured could affect heritability estimates. Thus, response to the vaccine may not be heritable (or lowly heritable) at the time points measured in this study, but heritability could be higher if different time points were evaluated.

Less phenotypic variance was observed in response to the booster vaccination, which may explain our low heritability estimates for the booster and overall response to the vaccine. Almost all animals vaccinated and sampled had a reasonably high humoral response to booster vaccination. Less phenotypic variance in the secondary response to vaccination suggests that calves had similar antibody responses to the booster vaccine, regardless of amount of antibodies produced in response to initial vaccine administration. While this result makes it difficult to quantify genetic differences in booster and overall humoral response, it does suggest that the booster vaccine is effective in eliciting antibodies. Alternatively, genetic variation of humoral response to this vaccine may affect to a greater degree animals’ primary immune response rather than secondary immune response. Indeed, cells and pathways involved in the primary and secondary immune responses do not completely overlap. Genes important for development of a primary immune response may not be relevant for development of a secondary immune response, and vice versa [21].

Previously published experiments investigating heritability of humoral response to vaccination often focused on the initial, primary response [22–24]. Heritability of antibody response to a Bovine Respiratory Syncytial Virus (BRSV) vaccine was estimated in Holstein and Charolais crossbred cattle following booster vaccine administration [3]. Response to this vaccine was evaluated at 14 days and 28 days after booster vaccination. Heritability estimates were highest at 14 days following booster vaccination (total BRSV IgG h^2^ = 0.29 ± 0.17), which was a time point not measured in our study. At 28 days after booster vaccine administration, heritability was lower but significantly different from zero (total BRSV IgG h^2^ = 0.11 ± 0.09). This time point is more similar to our experiment (35 days after booster vaccination). Our experiment may have uncovered more genetic variation in antibody response to booster vaccination if an earlier time point post-booster vaccination was measured. However, caution is warranted when comparing these studies because different vaccines were examined.

With the exception of antibodies present at day 21, heritability of our traits was not appreciably different between pedigree- and genomics-based methods of estimating genetic relationships. Hence while the ability to genotype thousands of SNP markers in cattle has made accurate estimation of relationship coefficients based on SNP genotypes feasible, and simulation studies have shown that heritability was estimated more accurately using genomic information as opposed to pedigree data [25], the method of estimation of relationship coefficients did not appreciably change most of our heritability estimates.

Our GWAS discovered eight SNPs that were associated with initial vaccine response, overall vaccine response, or both at the genome-wide significance level. Each of these SNPs were located in different areas of the genome; a “peak” of significant SNPs in a specific region of the genome was not observed in our study. Five of these SNPs were located within known or predicted protein coding genes; however, none of these genes have known function within the immune system. Several of these SNPs were highly significantly associated with initial and overall vaccine response: rs135467227 (found within *Myo16*) was the most significant SNP with a *P* < 1.56 x 10^−32^.

A total of 22 SNPs were associated at the genome-wide level with *E*. *coli* O157 antibodies present at day 0, the time of initial vaccination. The SNP effects on antibody titer at d 0 may be caused by genetic differences in colonization success among preweaning calves. Enterohemorrhagic *E*. *coli* induce the formation of pedestals on the surface of epithelial cells in lower gut of cattle. These pedestals facilitate tight binding of the bacteria to the epithelial surface and enable prolonged colonization. Host keratin filament and cytoskeleton genes may produce proteins that interact with *E*. *coli* O157:H7, and genetic variation in these genes may influence pedestal formation and successful colonization [15]. Alternatively, significant SNPs may be associated with genetic variation in passive antibody transfer from dam to calf. Although dams were never vaccinated with *E*. *coli* O157:H7, it is possible that previous exposure of dams to *E*. *coli* O157:H7 resulted in an antibody response, and these antibodies could be passively transmitted to their calves.

It is counter intuitive that we observed large SNP effects for antibodies present at d 0 yet heritability was near zero overall. In this case common sense or intuition are misleading because it is possible for regional heritability to be different from overall heritability if local relationship matrices differ by region and from genomic relationship averaged across the genome. Furthermore, the sum of variance contributed by multiple SNP can exceed overall heritability from discovery bias if the number of SNP exceeds the number of animals and if the SNP are not independent which is likely true because of linkage disequilibrium between nearby SNP. Estimability of heritability (regional or overall) depends on the variance in eigen-values of the genomic or pedigree relationship matrix. Populations with larger variances in eigen values yield more precise estimates of heritability [18]. The variance in eigen-values for whole genome relationship matrix was 1.34 for the genomic matrix and 1.13 for pedigree. Regional heritability could not and would not be estimated to be different from whole genome heritability if standardized regional genomic relationship matrices (whole chromosome or smaller segments) were nearly diagonal (little or no covariance between animals). In the case of nonrelatives or very distant relatives sampled from a breed with large effective population size formed many generations previous we would expect regional and overall relationship matrices to be equivalent. However, in our population we have close relatives and recent admixture from multiple breed sources so we expect regional and overall relationship matrices to be different and the variance in eigen values of standardized regional genomic relationship matrices to be greater than zero. Conversely, pedigree relationships represent an average over the whole genome so regional pedigree relationships are equivalent to overall and regional heritability is not identifiable or estimable independent of overall heritability with just pedigree information and no SNP genotypes. Nonzero variance in eigen values of regional genomic relationships standardized by whole genome relationships allows estimable differences in heritability by region and between region and the overall genome. The variance in eigen values for chromosome specific genomic relationship matrices standardized for whole genome relationships averaged 2.17 (ranged from 1.28 to 3.19) for the 29 autosomal chromosomes with the variance in eigen values decreasing with chromosome length. For smaller regions in the genome, the variance of eigen values of standardized relationship matrices increased; averaged 24.1 (ranged from 12.2 to 76.3) for 1,000 SNP regions (n = 762); averaged 38.1 (ranged from 18.3 to 127.9) for 500 SNP regions (n = 1,535), and averaged 60.6 (ranged from 24.8 to 234.4) for 250 SNP regions (n = 3,083).

It is also possible for functional SNP to not be in linkage disequilibrium with the observed SNP or with information from the pedigree if the functional SNP are rare and only segregating in small families; for example, if only a few of the dams are segregating and the sires are not.

Extrapolation of these data to other vaccines, species, and measures of vaccine response will require validation. The vaccine used in this experiment protects against *E*. *coli* O157 by generating host antibodies to SRP receptors on the surface of bacterial cells. Iron, essential for survival of *E*. *coli*, is taken up by *E*. *coli* O157 by these receptors and the presence of anti-SRP antibodies in intestinal mucosa prevents iron uptake, resulting in cell death. Thus, mechanism of action of this vaccine is different than most other vaccines in use. Further, vaccine response is specific to the adjuvant included in the vaccine preparation. Only humoral immune response, as measured by an ELISA specific for anti-SRP antibodies, was examined. Different heritability estimates and patterns of genome-wide association may be found if specific antibody subtypes, protective antibodies, or measures of innate or cell-mediated immune response were examined. We made no attempt to validate efficacy of this vaccine, which has previously been published [13], although results suggest that the booster vaccine is eliciting a humoral response.

Heritability of the initial humoral response to *E*. *coli* O157 vaccination was estimated to be 0.10–0.14, although these estimates were not statistically significant from zero. Further, SNPs associated with humoral response to this vaccine were identified. Prediction of breeding values for vaccine response could help identify bulls that do or do not respond robustly to commercially available vaccines. Response to vaccination is a complex trait. Timing of measurement of vaccine response will impact our ability to predict breeding values. How vaccine response is measured should also be considered before selection for vaccine response can be achieved. Selection on vaccine response could improve herd health. Calves predicted to have poor response to commercially available vaccines could be culled at an early age, resulting in improved herd health without the use of antibiotics or development of more protective vaccines.

## Supporting information

S1 Table Top 100 SNPs ranked by P-Value associated with each trait. Includes additive and dominance effects of each SNPs assuming 1) a common heritability for each SNP and 2) a simultaneous analysis, which allowed SNP effects to change heritability and environmental variance. Results obtained using a genomic relationship matrix and a pedigree relationship matrix are reported.
(XLSX)

S1 Fig Manhattan plot of genome-wide single nucleotide polymorphisms (SNPs) associated with antibodies present at time of initial vaccination (day 0) with *E*. *coli* O157:H7 Siderophore and Receptor Porin (SRP) proteins in calves (*n* = 651). Red line = Genome-Wide Significance Level (*P* < 5 x 10^−8^); Blue line = Suggestive Significance Level (*P* < 1 x 10^−5^). A genomic relationship matrix was used for this Manhattan Plot, but similar results were obtained using a pedigree relationship matrix (results not shown).
(TIFF)

S2 Fig Manhattan plot of genome-wide single nucleotide polymorphisms (SNPs) associated with antibodies present at time of booster vaccination (day 21) with *E*. *coli* O157:H7 Siderophore and Receptor Porin (SRP) proteins in calves (*n* = 651). Red line = Genome-Wide Significance Level (*P* < 5 x 10^−8^); Blue line = Suggestive Significance Level (*P* < 1 x 10^−5^). A genomic relationship matrix was used for this Manhattan Plot, but similar results were obtained using a pedigree relationship matrix (results not shown).
(TIFF)

S3 Fig Manhattan plot of genome-wide single nucleotide polymorphisms (SNPs) associated with antibodies present 56 days following booster vaccination with *E*. *coli* O157:H7 Siderophore and Receptor Porin (SRP) proteins in calves (*n* = 651). Red line = Genome-Wide Significance Level (*P* < 5 x 10^−8^); Blue line = Suggestive Significance Level (*P* < 1 x 10^−5^). A genomic relationship matrix was used for this Manhattan Plot, but similar results were obtained using a pedigree relationship matrix (results not shown).
(TIFF)

S4 Fig Manhattan plot of genome-wide single nucleotide polymorphisms (SNPs) associated with booster response (d 56 –d 21) to vaccination with *E*. *coli* O157:H7 Siderophore and Receptor Porin (SRP) proteins in calves (*n* = 651). Red line = Genome-Wide Significance Level (*P* < 5 x 10^−8^); Blue line = Suggestive Significance Level (*P* < 1 x 10^−5^). A genomic relationship matrix was used for this Manhattan Plot, but similar results were obtained using a pedigree relationship matrix (results not shown).
(TIFF)
