# Supplementary figures and images for: Genetic variation in humoral response to an Escherichia coli O157:H7 vaccine in beef cattle
Source: PLoS One. 2018 May 14;13(5):e0197347. doi: 10.1371/journal.pone.0197347 (PMC5951538; doi:10.1371/journal.pone.0197347)

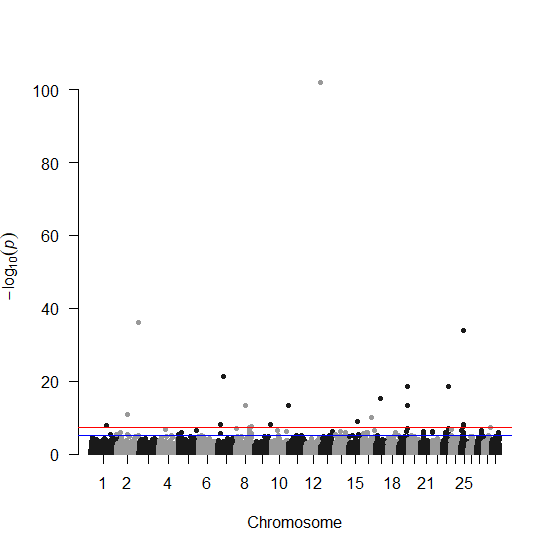

Supplement: S1 Fig — Red line = Genome-Wide Significance Level (P < 5 x 10^−8^); Blue line = Suggestive Significance Level (P < 1 x 10^−5^). A genomic relationship matrix was used for this Manhattan Plot, but similar results were obtained using a pedigree relationship matrix (results not shown). (TIFF) [file pone.0197347.s002.tiff]

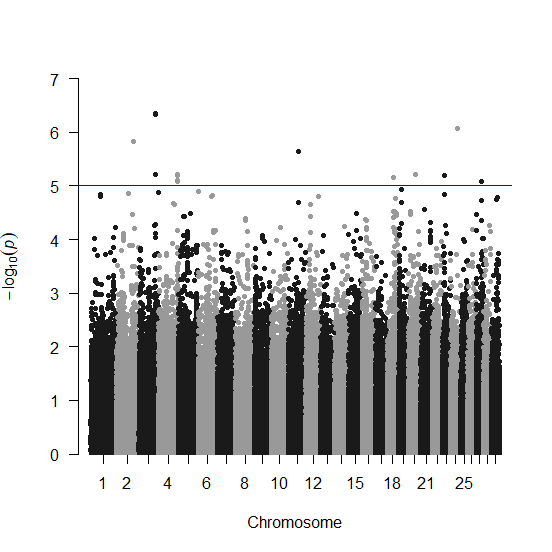

Supplement: S2 Fig — Red line = Genome-Wide Significance Level (P < 5 x 10^−8^); Blue line = Suggestive Significance Level (P < 1 x 10^−5^). A genomic relationship matrix was used for this Manhattan Plot, but similar results were obtained using a pedigree relationship matrix (results not shown). (TIFF) [file pone.0197347.s003.tiff]

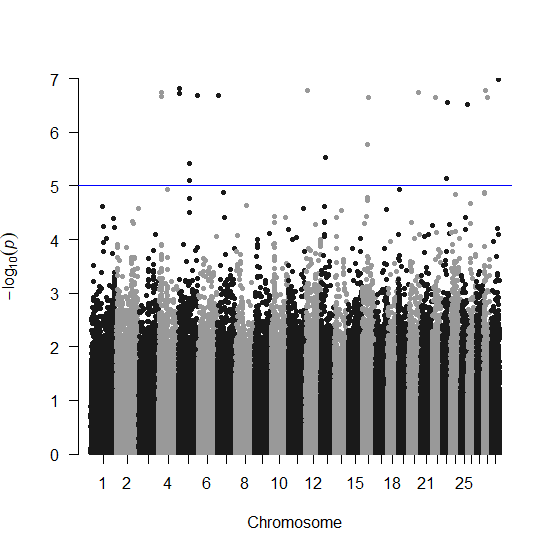

Supplement: S3 Fig — Red line = Genome-Wide Significance Level (P < 5 x 10^−8^); Blue line = Suggestive Significance Level (P < 1 x 10^−5^). A genomic relationship matrix was used for this Manhattan Plot, but similar results were obtained using a pedigree relationship matrix (results not shown). (TIFF) [file pone.0197347.s004.tiff]

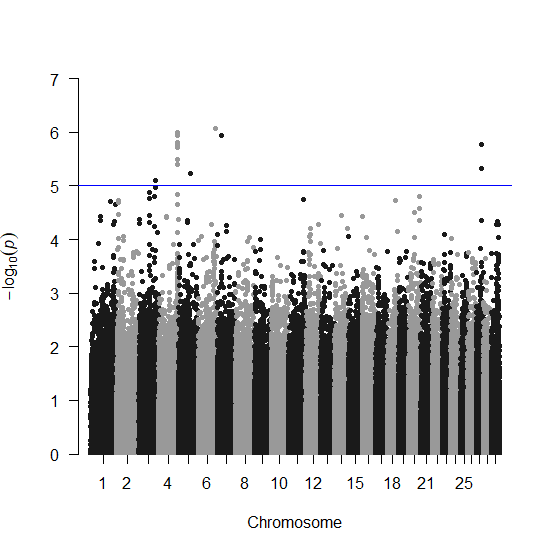

Supplement: S4 Fig — Red line = Genome-Wide Significance Level (P < 5 x 10^−8^); Blue line = Suggestive Significance Level (P < 1 x 10^−5^). A genomic relationship matrix was used for this Manhattan Plot, but similar results were obtained using a pedigree relationship matrix (results not shown). (TIFF) [file pone.0197347.s005.tiff]
